# Elucidation of the Glucose Transport Pathway in Glucose Transporter 4 via Steered Molecular Dynamics Simulations

**DOI:** 10.1371/journal.pone.0025747

**Published:** 2011-10-12

**Authors:** Aswathy Sheena, Suma S. Mohan, Nidhina Pachakkil A. Haridas, Gopalakrishnapillai Anilkumar

**Affiliations:** Amrita School of Biotechnology, Amrita Vishwa Vidyapeetham, Kollam, India; Universitat de Barcelona, Spain

## Abstract

**Background:**

GLUT4 is a predominant insulin regulated glucose transporter expressed in major glucose disposal tissues such as adipocytes and muscles. Under the unstimulated state, GLUT4 resides within intracellular vesicles. Various stimuli such as insulin translocate this protein to the plasma membrane for glucose transport. In the absence of a crystal structure for GLUT4, very little is known about the mechanism of glucose transport by this protein. Earlier we proposed a homology model for GLUT4 and performed a conventional molecular dynamics study revealing the conformational rearrangements during glucose and ATP binding. However, this study could not explain the transport of glucose through the permeation tunnel.

**Methodology/Principal Findings:**

To elucidate the molecular mechanism of glucose transport and its energetic, a steered molecular dynamics study (SMD) was used. Glucose was pulled from the extracellular end of GLUT4 to the cytoplasm along the pathway using constant velocity pulling method. We identified several key residues within the tunnel that interact directly with either the backbone ring or the hydroxyl groups of glucose. A rotation of glucose molecule was seen near the sugar binding site facilitating the sugar recognition process at the QLS binding site.

**Conclusions/Significance:**

This study proposes a possible glucose transport pathway and aids the identification of several residues that make direct interactions with glucose during glucose transport. Mutational studies are required to further validate the observation made in this study.

## Introduction

Glucose, the main source of energy for all eukaryotic cells, is transported by a class of polytopic membrane proteins called glucose transporters (GLUTs) [1], [2]. Among GLUTs, glucose transporter4 (GLUT4) is the major insulin facilitated glucose transporter, predominantly present in tissues such as adipose and muscle, which plays an important role in maintaining blood glucose homeostasis [3], [4]. This transporter protein comprises 12 transmembrane helices; an N-terminal domain of helices I-VI and a C-terminal domain of helices VII-XII. These two half bundles form a pseudo symmetry around a central polar tunnel that permeate glucose [5], [6].

Biochemical studies have been carried out in glucose transporters (especially GLUT1) to understand the sugar transport mechanism across these transporters. Studies on GLUT1 have revealed that transmembrane segments 1, 2, 4, 5, 7, 8, 10 and 11 form the glucose transport tunnel and its amphipathic nature suggests the possibility of an aqueous permeation pore for the glucose transport [7], [8], [9], [10], [11], [12], [13], [14]. Mutation of Gln298 (Gln282) (throughout the manuscript, the residue in bracket represents the corresponding residue in GLUT1) alters the sugar binding specificity and this is the single residue shown to be directly interacting with glucose [15]. Another residue, Gln177 (Gln161) is reported to be involved in the exofacial binding site [16]. Tryptophan residues at the endofacial binding site play a key role in glucose transport activity. Mutation at the Trp404 (Trp388) residue resulted in a reduced membrane targeting of GLUT4, whereas the Trp428 (Trp412) mutant exhibited a reduced intrinsic activity [17], [18]. Residues Ser310 (Ser294) and Thr311 (Thr295) are important for switching the transporter between inward and outward conformations [19]. In another study, the mutation of Tyr309 (Tyr293) locked the transporter in an outward conformation concluding that this residue is involved in tunnel gating after glucose entry [20].

A homology model of GLUT4 was generated in our laboratory to understand the various structural and functional aspects of this transporter, since no crystal structures were available for any of these family members [21]. The model was based on *E.coli* Glycerol 3-phosphate transporter and the biochemical data available on GLUT1. GLUT1 shows a sequence similarity of 63.3 % with GLUT4. The transmembrane regions between these transporters possess a higher identity while the differences reside in the N- and C-terminus and the loop regions. The variations in the loop, N- terminal motifs such as FQQI and the dileucine (LL) motif in the C-terminus account for the differences in regulation by hormones, tissue distribution and transport kinetics [22], [23], [24]. The model was validated by docking studies with known substrates (glucose) and inhibitors (genestein and cytochalasin B) [21], [25]. The interactions obtained from these docking studies were consistent with the experimental data. Furthermore, we were able to reveal the molecular mechanism of insulin stimulated glucose inhibitory action of Kaempferitin using this model [25]. Later, a conventional molecular dynamics study was carried out to explain the conformational rearrangements of GLUT4 in the presence of glucose and ATP [26]. This study also provided an explanation for ATP mediated glucose transport inhibition by GLUT4 [27], [28]. The complete translocation of glucose through the tunnel could not be accomplished in the time scale used for a conventional molecular dynamics simulation. However, SMD study can be used to study the movement of glucose through the tunnel. SMD studies were used successfully on many crystal structures and homology models of membrane transporter proteins to understand the energetics and sterioselectivity of substrate transport across the tunnel [29], [30]. Crystal structures of Major Facilitator Superfamily(MFS) members like GlpT and LacY were used for SMD simulation studies to reveal their substrate specificity and the transport mechanism [31], [32]. Gu *et.al* have used similar approach to explain the substrate permeation pathway for glutamate transporter and the significant role of Na+ ions in substrate transport [33]. Homology models of MscL, a mechanosensitive channel, and GlpF, aquaglyceroporin have also been subjected to SMD simulation studies to understand the substrate transport mechanism [34], [35].

In the present work, we have employed SMD technique to understand the glucose transport mechanism through the permeation pore of GLUT4. Multiple SMD experiments were employed to pull the glucose through the tunnel. These simulations revealed a possible pathway for glucose transport and identified significant interactions with the residues lining the pathway. This study proposes an atomic explanation for the specific roles of all the residues that have been shown to be involved in glucose transport process. Trajectory analysis proposed the role of hydrophobic patches at the tunnel entry and exit during glucose transport. Here, we propose an 180° rotation of glucose on its vertical axis near central sugar binding site that helps the sugar recognition process and its further navigation through the tunnel. The energetics analysis of glucose transport has revealed the transport mechanism as a three step process, *viz* substrate occlusion, translocation to the local binding sites and its release to the cytoplasm.

## Results and Discussion

**Figure 1:**
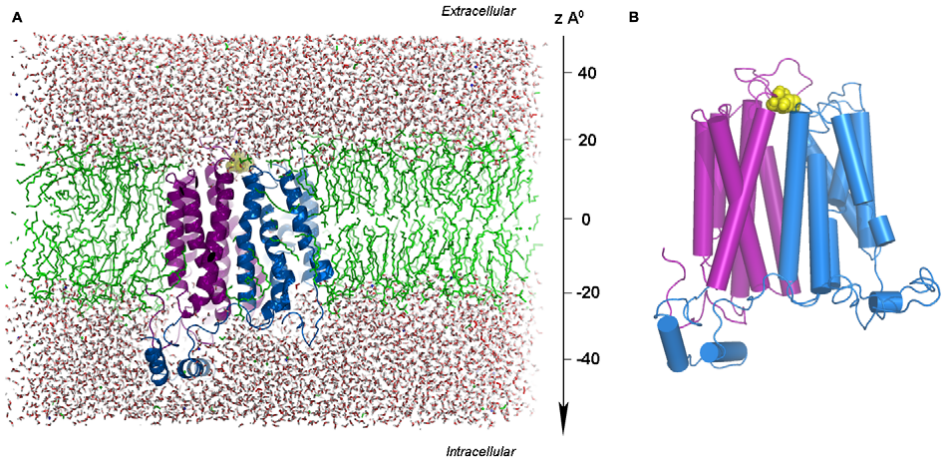
GLUT4-glucose complex embedded in the simulation box. (A) A side view of initial simulation box. GLUT4 (two domains shown in different color, TM1 to TM6 shown in violet and TM7-TM12 shown in marine blue) was inserted in the lipid molecules (green) and surrounded by water (red). Glucose molecule is shown in yellow spheres. (B) A close view of GLUT4 and glucose complex. The glucose bound at the entry of the channel is shown in yellow sphere.

The GLUT4 model embedded in the lipid bilayer was used as the input structure for the SMD in which the glucose bound at the extracellular face of the transporter has been pulled through the permeation pore applying a constant velocity pulling method along the z-axis (Figure 1). Since the glucose ring is flexible, it is highly probable that it may undergo conformational changes in the binding pocket, an equilibrium simulation of 1ns was performed and several snap shots were selected for multiple SMD experiments. Six SMDs were carried out with varying starting pose and the trajectory analyses showed that all the simulations followed a common pathway irrespective of the initial pose of glucose. A similar kind of observation was seen in the case of lactose permease where the ligand followed the same displacement path in all the SMDs started from equilibrium simulations [32]. Moreover, multiple SMD approaches have been successfully employed in the case of fatty acid transporter where majority of the pulling simulations followed the same pathway [36]. Likewise, the stable conformational changes of Na+ ions in the aquaporin channel was studied using multiple equilibrium SMDs [37]. These studies show that though the ligands may have multiple conformations for the entry to the channel it may ultimately follow a common path and the equilibration simulations will identify the most appropriate poses for SMDs. Two representative trajectories (hereafter termed as SM1 and SM2) of the common pathway were discussed in this paper.

**Figure 2:**
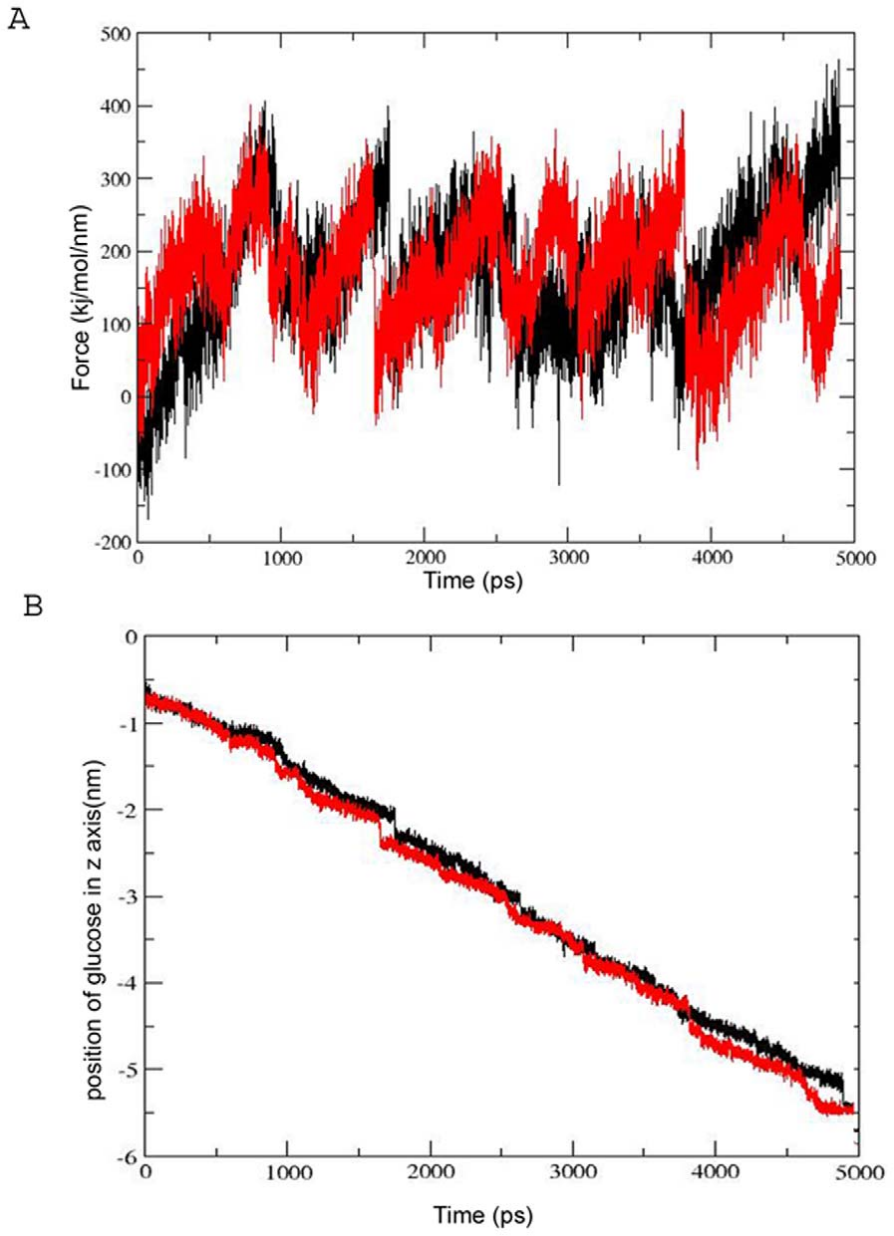
Force and displacement graph of common pathway. (A) Force profile during the transport of glucose through the channel. (B) Glucose permeation trajectory from extra cellular (+Z) to intracellular side (-Z) of the transporter. Black color represents the SM1 and grey color represents SM2.

From this study, it was revealed that one pose of the glucose closely followed the glucose transport model proposed by Barnett [38], [39]. In this pose (SM1), C4 and C6 of the glucose directed towards the extracellular face while C1 was facing the transporter tunnel. In the second pose (SM2), glucose maintained an orientation in which the ring oxygen and C1 were facing the exterior of the pore. These poses were subjected to simulation studies with different pull rates. At high pull rate (0.005 nm ps^−1^), there were differences in the trajectories and force profiles during the substrate permeation. However, these differences were not observed at the lower pull rate (0.001 nm ps^−1^) (Figure 2) and both SM1 and SM2 followed a common pathway irrespective of the starting pose of the glucose.

### Glucose transport mechanism

GLUT4 comprises a polar aqueous path between the N-terminal and C-terminal half of the protein through which glucose is carried to the cytoplasm. Several studies have suggested that the sugar hydroxyl group makes several hydrogen bond interactions with the tunnel residues thus facilitating the transport of glucose through this pore. In the present SMD analysis, it was possible to elucidate the various interactions of each of these residues among themselves and with individual functional groups of glucose that aid in its transport. Table S1 shows detailed analysis of all hydrogen bonds and hydrophobic interactions between glucose and transporter residues generated during the movement of glucose through the transport pathway. Using mutagenesis studies in GLUT1, several residues identified in this analysis were already shown to be involved in glucose transport pathway suggesting the validity of the present SMD study. In order to provide a better explanation for the entire glucose translocation pathway, the complete process was divided into three phases; movement of glucose from the tunnel mouth to the sugar binding site was considered as phase I, glucose at the sugar binding site was Phase II where glucose passes through the central sugar binding site and during Phase III glucose gets released into cytoplasm. In these SMD simulations, certain specific events in each of these phases were identified and considered as the keystone of the glucose transport process. A possible glucose transport mechanism was elucidated based on these observations and most of them were in agreement with the available biochemical studies.

#### I) Glucose entry to the tunnel

**Figure 3:**
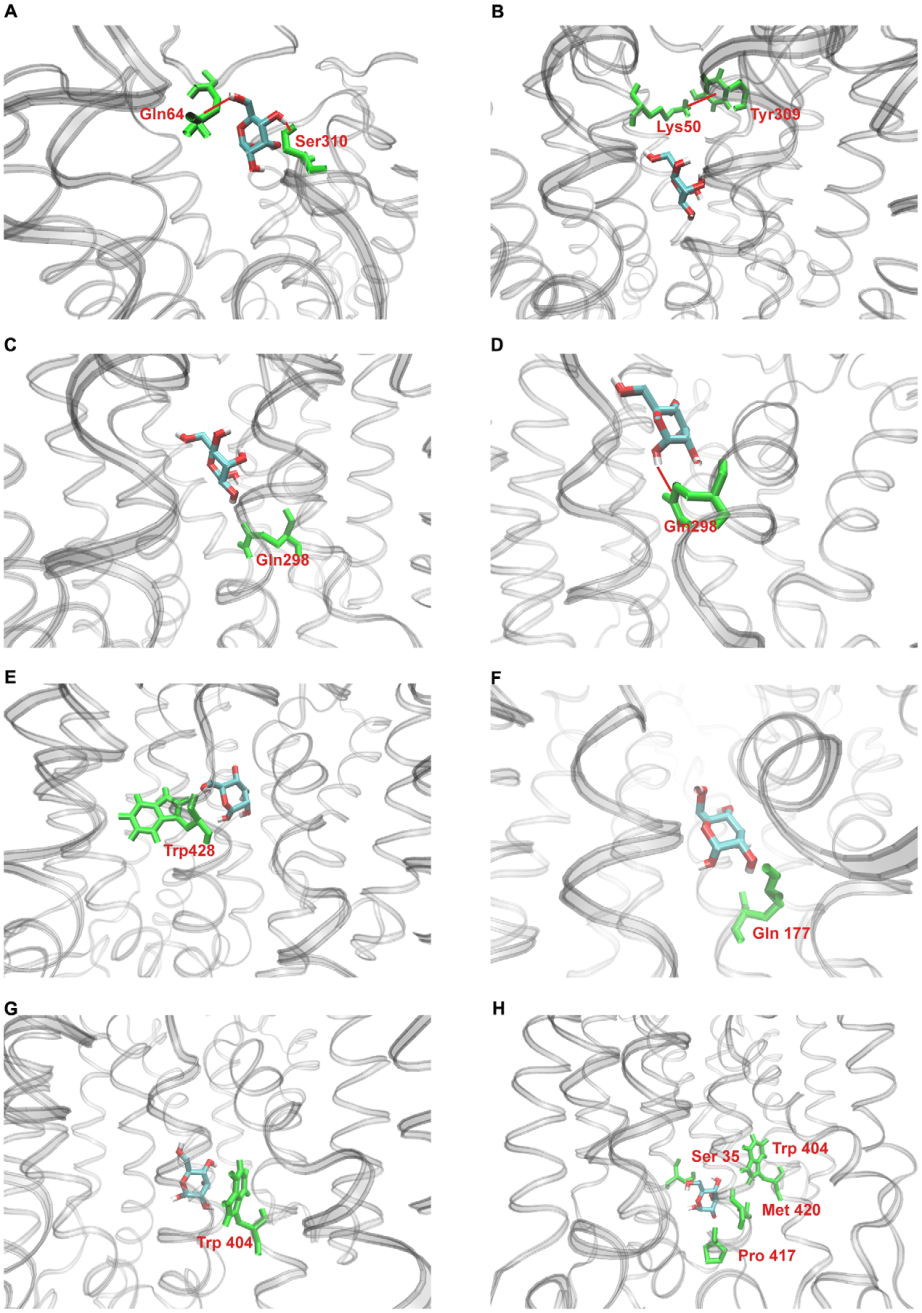
Glucose interaction with tunnel lining residues. (A) Initial interaction of glucose with Gln64 and Ser310 residues. Hydrogen bonding formed between C4-hydroxyl and Gln64 is represented by red dotted lines. (B) Tyr309-Lys50 mediated “gate” is closed behind the glucose molecule in the tunnel The thick red line represents the cation-pi interaction. (C and D) glucose rotated 180° in vertical axis. Rotation mediated the formation of a hydrogen bond between C1-hydroxyl of glucose and Gln298. (E) C6 hydroxyl of sugar molecule interacts with the Trp428 residue. (F) Hydrogen bond interaction between Gln177 and C3 hydroxyl of glucose. (G) Hydrophobic stacking of Trp404 and glucose ring. (The distance between the tryptophan ring and glucose ring during the transport was calculated) (H) Interaction between Met420 of TM11 and Trp404 of TM10 that blocks the permeation of glucose between these two helices.

**Figure 4:**
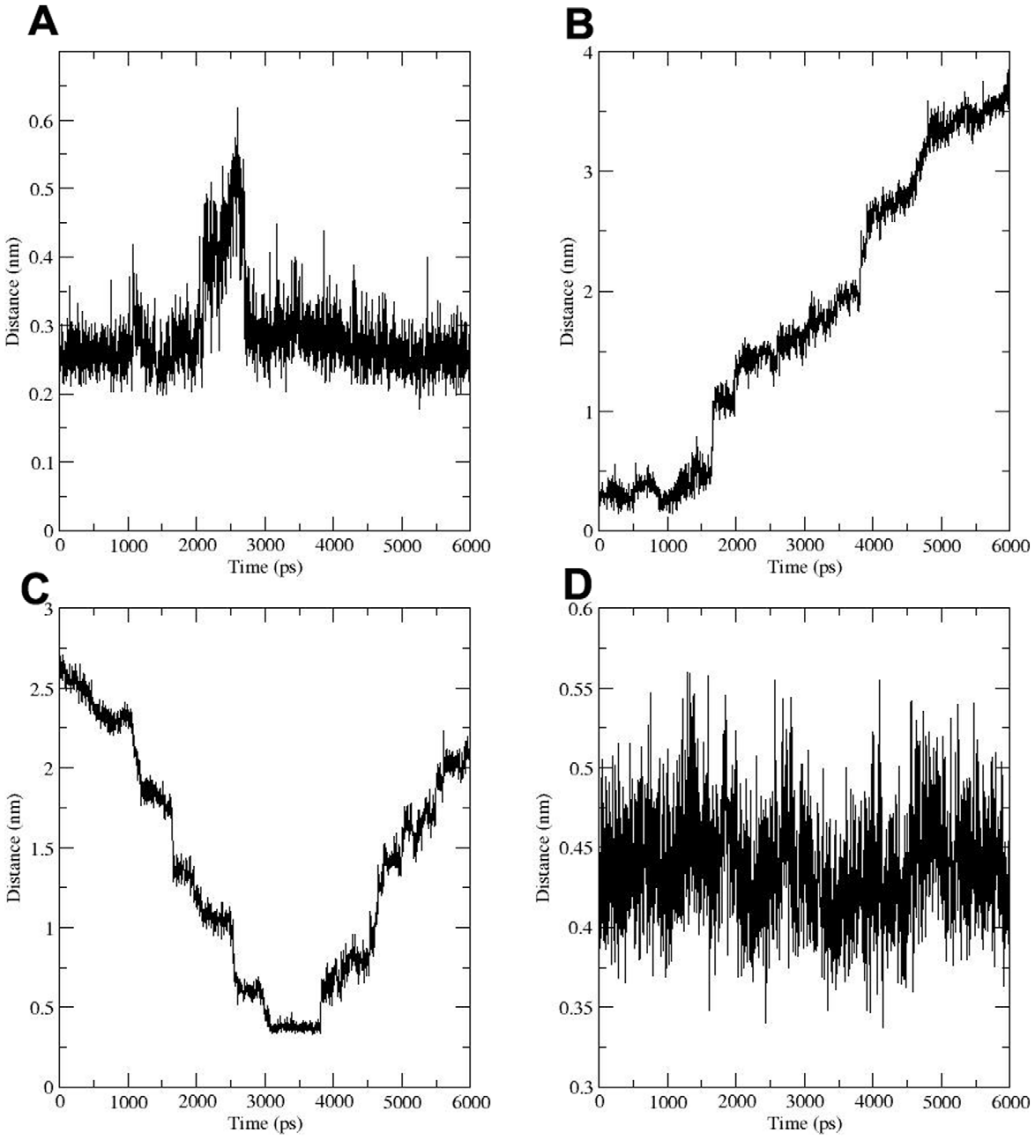
Distance plots for the glucose-residues, residue-residue interactions. Distance between (A) residues Tyr309 (aromatic ring) and Lys50 (NH3) that formed a cation-pi “gate”. B) C6 of glucose and the aromatic ring of Tyr309. (C) Hydrophobic stacking of glucose ring and the aromatic ring of Trp404. (D) Met420 and the aromatic ring of Trp404.

In every simulation, glucose was pulled from the tunnel mouth to the cytoplasm. Trajectory analysis showed that glucose entered the pathway anchoring C1, C2, C3 to the tunnel interior and C4 and C6 directed towards the extracellular face. This sugar orientation was similar to the experimentally proposed sugar transport model by Barnett et al. [38], [39]. The hydrogen bond interactions with the C1, C2 and C6 hydroxyls of sugar with Ser310, Gln64 and Tyr309 were important for attaining this pose (Figure 3A). The significance of Ser310 for the conformational rearrangement of the transporter is known [19]. However, so far no study has shown a role for this residue in the direct interaction with glucose. Furthermore, our study for the first time suggested a hydrogen bond interaction of Gln64 with substrate. Another critical observation in our SMD was the formation of a cation-pi interaction between Tyr309 and Lys50 at the tunnel entry resulting in the formation of an external gate. The hydrogen bond interactions between these residues and the C1, C2 and C6 hydroxyl of the sugar moiety facilitate the opening of this gate by disrupting this cation-pi interaction and thus gaining the entry of glucose into the pathway (Figure 3B). Once the glucose molecule crosses this “gate”, the cation-pi interaction between the residues is restored (Figure 4A). This interaction aids in occluding the sugar molecule within the transporter tunnel. It is interesting to note that a mutation of Tyr309 (Tyr293) to Ile inhibited the glucose transport whereas a substitution to Phe failed to produce any noticeable effect. Our SMD study provides a molecular explanation for the differential effect of two substitutions of the same residue on glucose uptake suggesting the requirement of an aromatic ring at this position.

Biochemical studies have identified a “hydrophobic patch” generated among Tyr307, Phe308 and Tyr309. It was suggested that the C6 carbon atom of glucose comes close to this hydrophobic patch and this orientation is important for the proper movement of glucose through the tunnel [20]. Our trajectory and distance plot analysis (Figure 4B) suggested that C6 of glucose comes close to Tyr309 and this tendency was seen in all six different poses selected from the equilibrium simulations supporting this biochemical data. This orientation of glucose was important for positioning the glucose in such a way that the hydroxyl groups at C1, C2 and C3 can now form hydrogen bonds with the polar residues (Asn46, Gln49, Gln439, Thr311) for facilitating its further movement to the sugar binding site. The residue, Thr311 (Thr295) is shown to be important for the glucose transport [19], while the importance of remaining residues, Asn46, Gln49 and Gln439 needs to be investigated.

#### II) Glucose at the sugar binding site

Several hydrogen bond interactions were formed between glucose (C1, C2, C3 hydroxyls) and amino acid residues (Val85, Ile45, Gly435) proximal to the sugar binding site. At this point, the residue Asn431 located in TM11 started interacting with the C2 sugar hydroxyl groups during its approach to the sugar binding site. This observation was supported by a biochemical study where mutation at Asn431 (Asn415) reduced the glucose transport activity [13]. Here, glucose underwent a rigid body rotation of 180° in its own vertical axis which aided to form a hydrogen bond between C1 hydroxyl of glucose and the Gln298 residue (Figure 3C and 3D). Previous studies have shown that the Gln298 of QLS motif was crucial for the binding of C1 hydroxyl of the sugar [15], [40]. In the next 500ps simulation, glucose passed the sugar binding site by the formation and breakage of hydrogen bonds with Gln177, Asn176, Ser301, Ser297 and the C1, C2 and C3 hydroxyls of glucose. In a previous mutagenesis study, this Gln177 (Gln161) was shown to reduce the specific activity of GLUT1 [10], [16]. Mutational studies are required to further confirm the role of Ser301, Ser297. The hydrophobic interaction between C6 and the Phe38 and Trp428 residues facilitated the polar contacts with these residues (Figure 3E and 3F). Thus, it can be concluded that the hydrophobic patches at the tunnel entry and below sugar binding site are responsible for positioning the glucose in the correct orientation and favoring the hydrogen bond interactions with the polar residues in the tunnel. Studies have clearly suggested an important role for the conserved Trp residues, Trp404 (Trp388) and Trp428 (Trp412) in glucose transport. Mutations of these residues significantly reduced the glucose transport [13], [17], [18]. However, these studies could not suggest a direct interaction of these Trp residues with glucose. Data generated from distance plot analysis suggests a possible hydrophobic interaction between C6 and the aromatic ring of Trp428 (Figure 4C).

#### III) Glucose release to the bulk solvent

The tunnel exit was lined by aromatic or hydrophobic residues Phe38 (TM1), Met420 (TM11), Trp404 (TM10). Visualization and distance plot analysis showed a hydrophobic stacking of the glucose ring and the aromatic ring of Trp404 residue (Figure 3G and Figure 4D). Apart from this stacking interaction, a hydrophobic interaction was also noticed between Trp404 and Met420 bringing TM10 and TM11 close to each other blocking the exit of glucose through this route (Figure 4D). As a result of these hindrances, glucose moved to the TM1, TM5 and TM11 via interactions with Ser35 (with C1 hydroxyl) Ala31 (with C4 hydroxyl) and Pro417 (with C2 hydroxyl) (Figure 3H). Strikingly, one previous study in GLUT1 has shown that mutation of Ser35 (Ser23) significantly reduced the glucose uptake [7]. This further confirms the role of this residue in TM1 in glucose exit.

### Sugar orientation in the GLUT4 tunnel

**Figure 5:**
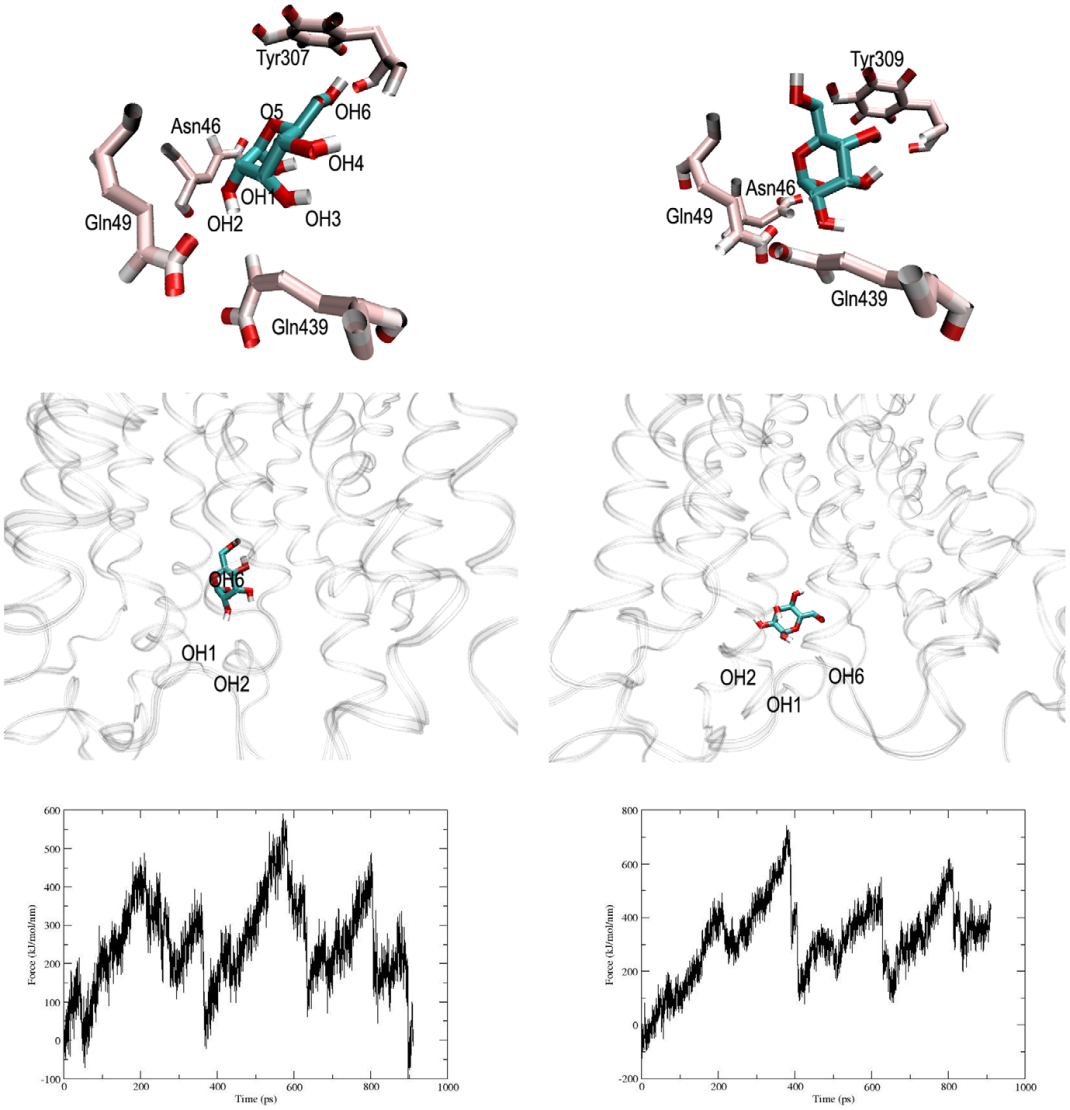
Sugar orientation of glucose in GLUT4 tunnel. (A) Snapshots from SM1 and SM2 (pulling velocity 0.005 nm ps^−1^), representing a favorable (left) and an unfavorable (right) orientation during glucose entry. (B) Glucose attained the proposed sugar model in the tunnel exit (left), while the orientation was not maintained in SM2 (right). (C) Required force profiles for SM1 (left) and SM2 (right) are shown here.

Glucose needs a particular orientation during the transport. In the present study, when SMD was carried out with a pull rate of 0.005 nm ps^-1^, the C6 of the glucose in SM1 was facing the extracellular face and this orientation facilitated a series of hydrogen bonds formation between the hydroxyls at C1, C2 of glucose and the polar residues (Asn46, Gln49, Gln439) in GLUT4 (Figure 5A , left). In this pose, a force of 400 kj mol^−1^ nm^−1^ was only needed to pull the glucose from this point down to the tunnel (Figure 5C, left). However, the glucose in SM2 was jerked at the similar pulling rate and attained a pose in which the C1, C2 hydroxyl groups were facing the hydrophobic residue, Tyr309 in the tunnel (Figure 5A, right). Due to this unfavorable interaction, it failed to make the necessary hydrogen bond interactions between C1, C2 hydroxyls and the polar residues, and therefore required a large pulling force (750 kj mol^−1^ nm^−1^) to move further (Figure 5C, right). Glucose in this pose make large number of constraints during the transport through the tunnel requiring large pulling force and was not supporting any biochemical or biophysical studies conducted so far to explain the sugar transport (Figure 5B, right). On the contrary, SM1 orientation of glucose was in full agreement with the model proposed by Barnett et al. for glucose transport (Figure 5B, left) [38], [39].

### Energetics of glucose transport

**Figure 6:**
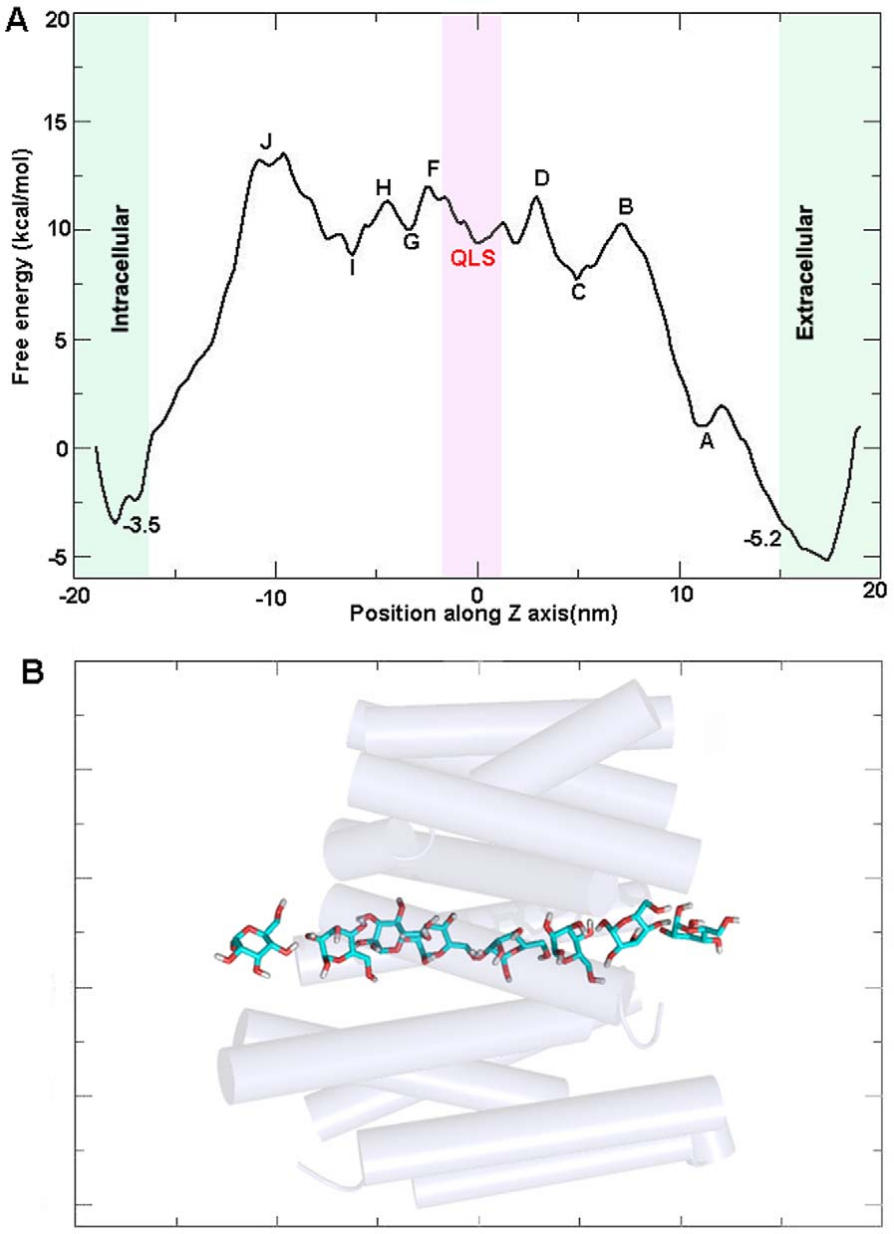
PMF profile and snap shots of glucose permeation. (A) PMF plot of glucose transport through the GLUT4 tunnel. The point A shows nearly zero energy where glucose attained the favorable orientation. The first highest energy (A to B in Fig A) barrier represents the glucose movement through the “gate” formed by Lys50 and Tyr309, via formation and breakage of several hydrogen bonds. Consecutive minima and maxima (from C to I) are due to the rotation of glucose and its passage through the QLS site. The point J represents the stacking of glucose with Trp404 and, breakage of hydrogen bonds with Asn176 and Gln177. The final large energy barrier (I to J) is due to the steric barrier introduced by the hydrophobic residues (Trp404 and Met420) in the tunnel and the reduced pore size. (B) Selected positions of glucose along the transport tunnel. Helices are shown as transparent cylindrical helices and glucose is shown in cyan sticks (with oxygen in red and hydrogen in white).

Using umbrella sampling technique, PMF (free energy) of glucose transport through the tunnel was calculated (Figure 6A). The PMF plot showed several consecutive small local energy minima and maxima with relatively large energy barriers at the entry and exit of the tunnel. Initial plateau near the 12 A° (A in Figure 6) of the tunnel corresponds to the rearrangement of glucose to achieve a favorable orientation. The highest free energy barrier (A and B in Figure 6) of the PMF accounts for the multiple events that facilitated the movement of glucose through the external gate generated via cation-pi interaction, hydrophobic patches and breakage of several polar contacts. In PMF, the transition of energy minima (C to I in Figure 6) corresponds to the formation and breakage of hydrogen bond interactions as discussed in PhaseI and PhaseII. The QLS site was located in Phase II and a corresponding energy barrier or well was absent in our PMF. At both QLS and neighboring regions, the force encountered by glucose in the trajectory was mainly due to the hydrogen bonds. Since similar kind of interactions were involved at QLS and neighboring regions, it is possible that the PMF variations observed are not drastically different in these regions, though we observed a small variation between QLS and neighboring regions. It is not surprising to see such a PMF pattern. Jensen et. al reported a similar PMF profile where an appreciable change in PMF was not detected between lactose binding site and its neighboring regions in the case of lactose transport analysis across the pathway of lactose permease [32]. Finally, at the glucose exit point, the PMF showed an energy barrier of ∼5 kCal mol^−1^ (I to J in Figure 6). This high energy barrier can be accounted by the hydrophobic hindrance formed by Trp404 and Met420 at the tunnel exit as well as the polar interaction of glucose at the exit with residues such as Ser35 of TM1 and Pro417 of TM11. In brief, our PMF profile represents the glucose transport as a three step process, substrate occlusion, translocation to the local binding sites and its release to the cytoplasm. Representative snap shots for the conduction of glucose taken for the PMF calculations are shown in Figure 6B.

The activation energy of the basal glucose transport activity in fat cells has been shown to be 10.5 kcal mol^−1^ [41]. It is interesting to note that the PMF calculation from our studies for the transport of glucose showed a 10 kcal mol^−1^ as the highest energy barrier. Further studies need to be carried out to corroborate the value as we have used a homology model for this SMD study. Here the predicted activation energy and overall free energy plot indicates the need of energy for the glucose transport, probably expended by the substrate gradient.

### Conclusions

**Figure 7:**
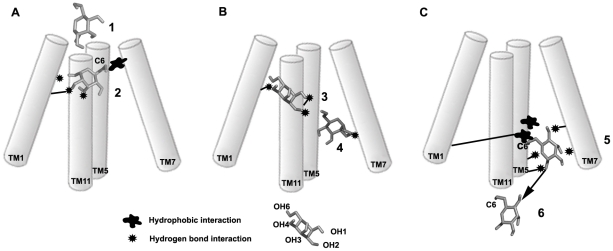
Schematic representation of glucose transport. (A) Major events in Phase I. Positioning of hydroxyl group at C6 of glucose directing towards tunnel exterior (1) C6 hydrophobic interaction with Tyr309 mediates the hydrogen bond interactions of C1, C2, C3 hydroxyls with the polar residues, Asn46, Gln49 and Gln439 (2). (B) Major events in Phase II. The rotation of glucose near the QLS site mediated by the hydrogen bonds with the residues Gly435(TM11), Gly35 (TM1), Asn 431 (TM11), and the C1 hydroxyl interacts with the Gln298 residue (3) and (4). (C) Anchoring of C6 to the Phe38 and Trp428 by a hydrophobic contact mediates hydrogen bond interactions with Asn176, Gln177 (5). Glucose released to the solvent near TM5 and TM11 (6).

SMD studies were used to delineate the pathway of glucose transport through the tunnel. This study identified a favorable orientation of glucose wherein C6 carbon atom of glucose was facing the extracellular face of the tunnel. This orientation aided glucose in aligning its C6 carbon with the hydrophobic patch seen at the transport pathway entry which was important to have further polar contact with other residues in the tunnel (Figure 7A). A rigid body rotation of substrate near the centre of the tunnel was required for the sugar recognition at the QLS site (Figure 7B). When it passed from the exofacial binding site, C6 of glucose came in contact with the aromatic residues Trp428 and Phe38 and this facilitated the polar interactions with Ser297, Ser310, Gln166 and Gln177 residues (Figure 7C). A constant interaction between Trp404 and Met420 redirected the glucose molecule to the TM1, TM5 and TM11, and dropped to the bulk solvent mediated by the interactions with Ser35, Pro417 and other loop residues. The highest energy barrier of 10 kcal mol-1 was required for the glucose occlusion which is in agreement with the calculated activation energy, the accuracy of this need to be corroborated with multiple approaches especially when the SMD was carried out with a well validated homology model. This study identified several residues that are already shown to have an effect in glucose transport by various biochemical studies. Additional mutational studies are needed to confirm the role of a few novel residues that were identified in our SMD studies involved in sugar transport.

## Materials and Methods

### Simulation System Setup

The starting structure used for this work was our GLUT4 model based on homology modeling study [21]. Glucose molecule was docked at the tunnel exterior of the GLUT4 model using the GLIDE program [42]. The protein preparation was done using the protein preparation wizard option provided by the GLIDE program. The glucose structure is optimized using the LigPrep module in the GLIDE program. Initially a Standards Precision (SP) docking was performed without setting any constraints. All other parameters of GLIDE were kept at default values. Extra Precision (XP) docking was then performed with the SP docking result to identify the best pose from the initial docking. Favorable binding pose was selected based on the GLIDE score and Emodel. The tunnel was solvated using the genbox program of GROMACS [43]. The tunnel was equilibrated with water using successive minimization and equilibration. Again the entire system was equilibrated for 4 ns followed by an energy minimization of 5000 steps using the Steepest Descent algorithm. The minimized docked complex (GLUT4-glucose) was embedded in a pre-equilibrated POPC (1-palmitoyl 2-oleoyl-sn-glycero-3-phosphatidylcholine) bilayer system placed in the solvated box with the help of visual molecular dynamics (VMD) [44]. The entire system was neutralized with an ion concentration of 0.15 M NaCl. We conducted a 1 ns equilibrium simulation with this system and from that six different glucose poses were selected for multiple SMDs. SMDS were performed with different pull rates of 0.005 nm ps^−1^and 0.001 nm ps^−1^ for all the six initial poses. The SMDs were repeated twice to check the consistency.

All simulations were carried out using GROMACS4.0.4 program with gmx force field. Electrostatic interactions were calculated using particle mesh Ewald method with a distance cut-off of 0.9 nm [45]. Lennard-Jones potential was used for describing the short range attractive and repulsive dispersion interactions with 1 nm cut-off. The solute, lipid and water were coupled using Berendsen temperature coupling with a temperature equal to 310 K [46]. A constant pressure was maintained using semi-isotopic pressure coupling (1 bar). The time step for integration was 1 fs and all bonds were constrained using LINCS algorithm [47].

We used a COM pulling method of GROMACS to pull the glucose molecule through the tunnel. Two different snap shots were used for running SMDs. A soft elastic spring of 1,000 kJ mol^−1^ nm^−2^ was attached to the glucose molecule and applied in the direction of +/-Z axis with a constant velocity. One of the residues at the extracellular side of the transporter was considered as the immobile reference in the pulling simulation.

### Umbrella sampling and PMF calculation

The average force experienced by the glucose all over the tunnel (PMF) was calculated using Umbrella Sampling and WHAM techniques [48], [49]. We generated a series of configurations along the reaction coordinate from the SMD experiments with a window spacing of 0.1 nm. To cover a range of −20≤z≤20 Å, total 45 windows (average of all SMD experiments) were selected. Each frame was sampled for 800 ps with a harmonic restraint of 1000 kJ mol^−1^ nm^−2^ applied on the glucose molecule. Final 500 ps were used for calculating PMF using the weighted histogram analysis method (WHAM) included in GROMACS as the g_wham utility. The PMF was calculated in kcal mol^−1^.

## Supporting Information

Table S1 **Hydrogen bonding and hydrophobic interactions with tunnel lining residues.** A detailed list of hydrogen bonding and hydrophobic interactions of sugar molecule with the tunnel residues of the transporter. To calculate the hydrogen bonds, a distance cut off of 3.5 A0 and an angle cut off 300 was used. A distance cut-off of < = 3 A0 between any non hydrogen atom of sugar and ring of residues Trp, Tyr and Phe was used for the calculation of hydrophobic interactions. Position represents the location of the bond forming residue in the transporter. Residues shown as underlined bold are those biochemically demonstrated to play a role in glucose transport (in GLUT1). These interactions were consistent in all SMD trajectories.
(DOC)
