# Supplementary material for: Elucidation of the Glucose Transport Pathway in Glucose Transporter 4 via Steered Molecular Dynamics Simulations
Source: PLoS One. 2011 Oct 12;6(10):e25747. doi: 10.1371/journal.pone.0025747 (PMC3192114; doi:10.1371/journal.pone.0025747)
Supplement: Table S1 — Hydrogen bonding and hydrophobic interactions with tunnel lining residues. A detailed list of hydrogen bonding and hydrophobic interactions of sugar molecule with the tunnel residues of the transporter. To calculate the hydrogen bonds, a distance cut off of 3.5 A0 and an angle cut off 300 was used. A distance cut-off of < = 3 A0 between any non hydrogen atom of sugar and ring of residues Trp, Tyr and Phe was used for the calculation of hydrophobic interactions. Position represents the location of the bond forming residue in the transporter. Residues shown as underlined bold are those biochemically demonstrated to play a role in glucose transport (in GLUT1). These interactions were consistent in all SMD trajectories. (DOC) [file pone.0025747.s001.doc]

**Table S1**

| **Position** | **Residue** | **Atom** | **z** | **References** |
| --- | --- | --- | --- | --- |
| Loop TM1-TM2 | Q64 | OE1, O, NE2 | 1.94 |  |
| Loop TM7-TM8 | **S310** | OG | 1.60 |  |
| Loop TM7-TM8 | **T311** | N, OG1 | 1.4 |  |
| Loop TM7-TM8 | **Y309** | OH , O | 1.34 |  |
| Loop TM1-TM2 | K50 | NZ | 1.27 |  |
| Loop TM11-TM12 | Q439 | NE2 | 1.25 |  |
| TM11 | G435 | N | 1.1 |  |
| Loop TM1-TM2 | Q49 | OE1, NE2, O | 0.97 |  |
| TM11 | G435 | O | 0.86 |  |
| Loop TM1-TM2 | N46 | OD1, ND2 | 0.619 |  |
| TM1 | Ile45 | O | 0.52 |  |
| TM7 | G302 | N | 0.45 |  |
| TM11 | **N431** | O, ND2 | 0.15 |  |
| TM7 | S301 | OG | 0.11 |  |
| TM7 | **Q298** | OE1, O | -0.26 |  |
| TM11 | **W428** | N, NE1, O | -0.36 |  |
| TM7 | S297 | OG | -0.38 |  |
| TM11 | **N427** | O, OD1, ND2 | -0.59 |  |
| TM5 | **Q177** | OE1, NE2 | -0.61 |  |
| TM5 | N176 | OD1, ND2, O | -1.09 |  |
| TM10 | **W404** | NE1 | -1.13 |  |
| TM1 | **S35** | N, OG | -1.30 |  |
| TM11 | M420 | O | -1.61 |  |
| TM1 | A31 | O | -1.80 |  |
| LoopTM10-TM11 | P417 | O | -2.00 |  |
| Loop TM4-TM5 | P165 | O | -2.62 |  |
| Loop TM4-TM5 | H167 | NE2 | -2.91 |  |
| **Hydrophobic Interactions** | | | |  |
| **Position** | **Residue** | **Atom** | **z** |  |
| TM7 | F307 | - | 0.89 |  |
| Loop TM7-TM8 | **Y309** | - | 1.34 |  |
| TM11 | **W428** | - | -0.36 |  |
| TM1 | F38 | - | -0.78 |  |
| TM10 | **W404** | - | -1.13 |  |
